# Role of 2D strain in the early identification of left ventricular dysfunction and in the risk stratification of systemic sclerosis patients

**DOI:** 10.1186/1476-7120-11-6

**Published:** 2013-02-03

**Authors:** Maurizio Cusmà Piccione, Concetta Zito, Gianluca Bagnato, Giuseppe Oreto, Gianluca Di Bella, Gianfilippo Bagnato, Scipione Carerj

**Affiliations:** 1 Clinical and Experimental Department of Medicine and Pharmacology – Cardiology, University of Messina, Via Consolare Valeria, A.O.U. Policlinico “G. Martino”, Messina, 98100, Italy; 2 Department of Rheumatology, University of Messina, Messina, Italy

**Keywords:** Systemic sclerosis, Echocardiography, Left ventricular dysfunction, 2D strain, Prognosis

## Abstract

**Background:**

Systemic sclerosis (SSc) is an autoimmune chronic disease characterized by diffuse fibrosis involving several organs, including heart. Aim of our study was to analyze left ventricular (LV) myocardial deformation, by use of 2D strain, in asymptomatic SSc patients with normal LV ejection fraction.

**Methods:**

We enrolled 29 SSc patients (28 female, 65±4 years) and 30 controls (23 female, 64±2 years). Echocardiographic study with tissue Doppler imaging (TDI) and 2D strain analysis was performed; moreover, patients were submitted to a two-year follow-up for the occurrence of cardiovascular events.

**Results:**

Standard echocardiographic parameters and TDI velocities were comparable between groups. LV longitudinal (LS) and circumferential (CS) strains were lower in patients than in controls (−13.1±4.8 *vs −*22.6±4.1, p < 0.001; -15.3±6.2 *vs −*20.4±5.6, p = 0.001), whereas radial strain (RS) was comparable between groups; moreover, a significant correlation of LS and CS with serum levels of Scl-70 antibodies was found (r = 0.74, p = 0.001; r = 0.53, p = 0.025). In addition, patients with cardiovascular events during follow-up showed a greater impairment of LS and CS (−10.3±2.5 *vs −*14.4±4.1, p = 0.015; -14.2±3.1 *vs −*20.1±1.6, p = 0.048) and higher values of Scl-70 antibodies serum levels (p = 0.047).

**Conclusion:**

The impairment of LV function, often subclinical, worsens prognosis of SSc patients, leading to increased risk of cardiovascular complications. 2D strain, allowing the early detection of LV abnormalities and the identification of patients at greater cardiovascular risk, may be a useful tool in order to provide a more accurate management of SSc patients.

## Background

Systemic sclerosis (SSc) is an autoimmune disease characterized by inflammation, widespread vascular lesions and fibrosis involving various tissues and organs such as skin, lungs, gastrointestinal tract, kidneys, heart and blood vessels [1-3]. Multiple cardiac abnormalities, including ventricular arrhythmias, conduction disturbances, pericardial effusion, myocardial fibrosis and ischaemia, have been, frequently, reported in SSc and associated to a worse clinical course of the disease [4]. In particular, right ventricular (RV) dysfunction, commonly associated with pulmonary hypertension, has been, traditionally, considered as the hallmark of cardiac involvement in SSc, whereas left ventricular (LV) involvement, confirmed at post-mortem examinations, has been less extensively evaluated [5,6]. Since LV abnormalities are often subclinical and detectable by standard echocardiography only in the advanced stage of the disease, the assessment of myocardial deformation, by use of two-dimensional (2D) strain, may be particularly valuable in the identification of early LV changes in SSc, allowing a more adequate management of these patients with particular regards to the prevention of overt cardiovascular complications.

Aim of the present study was to evaluate LV myocardial deformation, by use of 2D strain, with particular regards to its impact on clinical outcome, in asymptomatic SSc patients with preserved LV ejection fraction (EF).

## Methods

### Study population

We enrolled 29 consecutive asymptomatic patients (28 female, mean age 65±4 years) affected by SSc and 30 healthy subjects (23 female, 64±2 years). All patients fulfilled the criteria for SSc proposed by the American College of Rheumatology [7] and were affected by diffuse form of the disease with a time of 15±8 years from diagnosis. The therapeutic regimen employed from the onset of disease’s symptoms/signs, was, in all patients, the association of prednisolon and prostaglandin E1 (alprostadil). Pulmonary involvement was investigated in each patient by means of chest radiograph and computed tomography scan in order to detect interstitial fibrosis. Clinical examination, serological tests and echocardiographic study were performed in each subject. Furthermore, follow-up information was obtained from telephonic interviews with patients and controls, their relatives or their physicians every six months up to two years. Predefined end-points for cardiovascular events were the new onset of symptoms and/or signs of heart failure and coronary artery disease, atrial fibrillation or cardiovascular death during follow-up. Patients with arterial hypertension (SBP/DBP > 140/90 mmHg), documented coronary artery disease, moderate to severe valvular heart disease, regional myocardial asynergies, impaired LV EF (< 55%), atrial fibrillation, renal insufficiency (defined in case of creatinine clearance less than 90 ml/min/1.73 m^2^) and significant pulmonary interstitial fibrosis were excluded. Subjects with inadequate acoustic window were also ruled out (4 patients were excluded). All patients gave written informed consent to our study protocol that was supported by our hospital’s Ethical Committee. The authors of this manuscript have certified that they comply with the Principles of Ethical Publishing in the International Journal of Cardiology [8].

### Echocardiographic study

All echocardiographic examinations were performed with a My-Lab 50 echocardiographic machine (Esaote, Florence, Italy), equipped with a 2.5-MHz phased array transducer. Two-dimensional images were obtained from standard views (parasternal long-axis and short axis views, apical 4-chamber, 2-chamber and long-axis views). In each subject LV end-diastolic (EDV), end-systolic (ESV) volumes and biplane Simpson’s EF were measured. LV mass was calculated, according to Devereux’s formula, by M-mode technique, from mid-ventricular short axis view and corrected for body surface area [9]. Qualitative assessment of regional wall motion was performed according to the 16-segments model of the American Society of Echocardiography [10]. Mitral inflow measurements were obtained from apical 4-chamber view by placing the sample volume at mitral leaflet tips, and peak velocities of E and A waves, E/A ratio and E-wave deceleration time were measured. RV end-diastolic diameters were measured, as recommended by the American Society of Echocardiography, in parasternal short axis (RVOT Prox) and apical 4-chamber views at basal level (RVD1) [11]. Tricuspid annular plane systolic excursion (TAPSE) was measured with M-mode cursor positioned at the free wall angle of the tricuspid valve annulus; furthermore, RV fractional area change (FAC), defined as (end-diastolic area – end-systolic area)/end-diastolic area × 100, was calculated from apical 4-chamber view. Tricuspid valve regurgitation peak velocity, measured by use of continuous-wave Doppler technique, was employed to estimate systolic pulmonary arterial pressure (sPAP).

Tissue Doppler imaging (TDI) myocardial velocities were measured in the apical 4-chamber view, placing the sample volume at the junction of LV interventricular septum with mitral annulus. Peak systolic (s’), early (e’) and late diastolic (a’) myocardial velocities were recorded and E/e’ ratio was calculated.

In each subject, strain analysis was performed offline by use of a dedicated software package (XStrain TM, Esaote, Florence, Italy). With this software, already validated in previous studies [12,13], segmental evaluation of strain was performed from digitalized 2D videoclips of basal, mid-ventricular, apical parasternal short axis and of 4- and 2-chamber apical views. Endocardial border was manually traced as a sequence of 13 equidistant points and frame-by-frame displacement of these points was automatically evaluated. Global and segmental values of radial, circumferential (subendocardial layer) and longitudinal strains (RS, CS and LS respectively) were obtained. Tracking quality was verified for each segment and low quality images were excluded from strain analysis.

### Statistical analysis

Data were expressed as mean ± SD. Statistical analysis was performed using the SPSS statistical software (SPSS v.17 for Windows, SPSS. Inc., Chicago, IL, USA). Independent T and Chi-square tests were used for comparison between groups of continuous and categorical variables, respectively. Pearson’ and Spearman’s coefficients were used for correlations between variables. To assess the reproducibility of measurements of myocardial strains, Bland-Altman analysis was performed for intra-observer and inter-observer variability. P-values of 0.05 or less were considered significant.

## Results

General characteristics and prevalence of cardiovascular risk factors of SSc patients and healthy controls are shown in Table 1. There were no significant differences between patients and controls regarding age, systolic/diastolic blood pressure and prevalence of main cardiovascular risk factors with the only exception of sex because of a greater prevalence of female gender in SSc group (p = 0.03). As shown in Table 2, the two groups did not differ from each other with respects to LV mass, EDV, ESV, EF, Doppler mitral diastolic velocities and E-wave deceleration time as well as to RV diameters, TAPSE and FAC; differently, sPAP was mildly increased in patients with respects to controls (p < 0.001).

**Table 1:** General characteristics in controls and patients

	**Controls (n=30)**	**SSc (n=29)**	**p**
**Age, yrs**	64±2	65±4	ns
**Female sex, n (%)**	23 (76)	28 (96)	0.03
**SBP, (mmHg)**	120±11	125±15	ns
**DBP, (mmHg)**	79±8	74±9	ns
**Diabetes, n (%)**	3 (10)	3 (10)	ns
**Dyslipidemia, n (%)**	10 (33)	10 (34)	ns
**Smoking, n (%)**	3 (10)	2 (6)	ns
**Disease’s duration, yrs**	-	15±8	-
**Scl-70 antibodies, n (%)**	-	17 (58)	-
**Serum levels of Scl-70 antibodies, U/ml**	-	46.9±41.2	-

*SBP and DBP*: systolic and diastolic blood pressure, respectively.

**Table 2:** Standard echocardiographic parameters in controls and patients

	**Controls (n=30)**	**SSc (n=29)**	**p**
**LV EDV, ml**	79±11	83±15	ns
**LV ESV, ml**	27±7	33±14	ns
**LV EF, %**	65±1	64±6	ns
**LV Mass, g/m**^**2**^	68.9±5.9	69.6±4.6	ns
**Mitral E peak velocity, cm/s**	64±9.5	58.5±12.4	ns
**Mitral A peak velocity, cm/s**	70±9.2	66.3±14.2	ns
**Mitral E/A ratio**	0.8±0.3	0.8±0.1	ns
**Mitral E deceleration time, ms**	188.1±43	180.7±28	ns
**RVOT Prox, mm**	29.9±5.6	30.2±3.7	ns
**RVD1, mm**	33.1±2.9	33.1±2.9	ns
**TAPSE, mm**	22.5±3.1	21.6±2.9	ns
**RV FAC, %**	48.5±6.9	46.9±6.3	ns
**sPAP, mmHg**	21.8±6	37.4±9	< 0.001

*LV*: left ventricular; *EDV* and *ESV*: end-diastolic and end-systolic volumes, respectively; *EF*: ejection fraction; *RVOT prox*: right ventricular outflow tract proximal diameter; *RVD1*: right ventricular inflow diameter at basal level; *TAPSE*: tricuspid annular plane systolic excursion; *FAC*: fractional area change; *sPAP*: systolic pulmonary arterial pressure.

**Figure 1:**
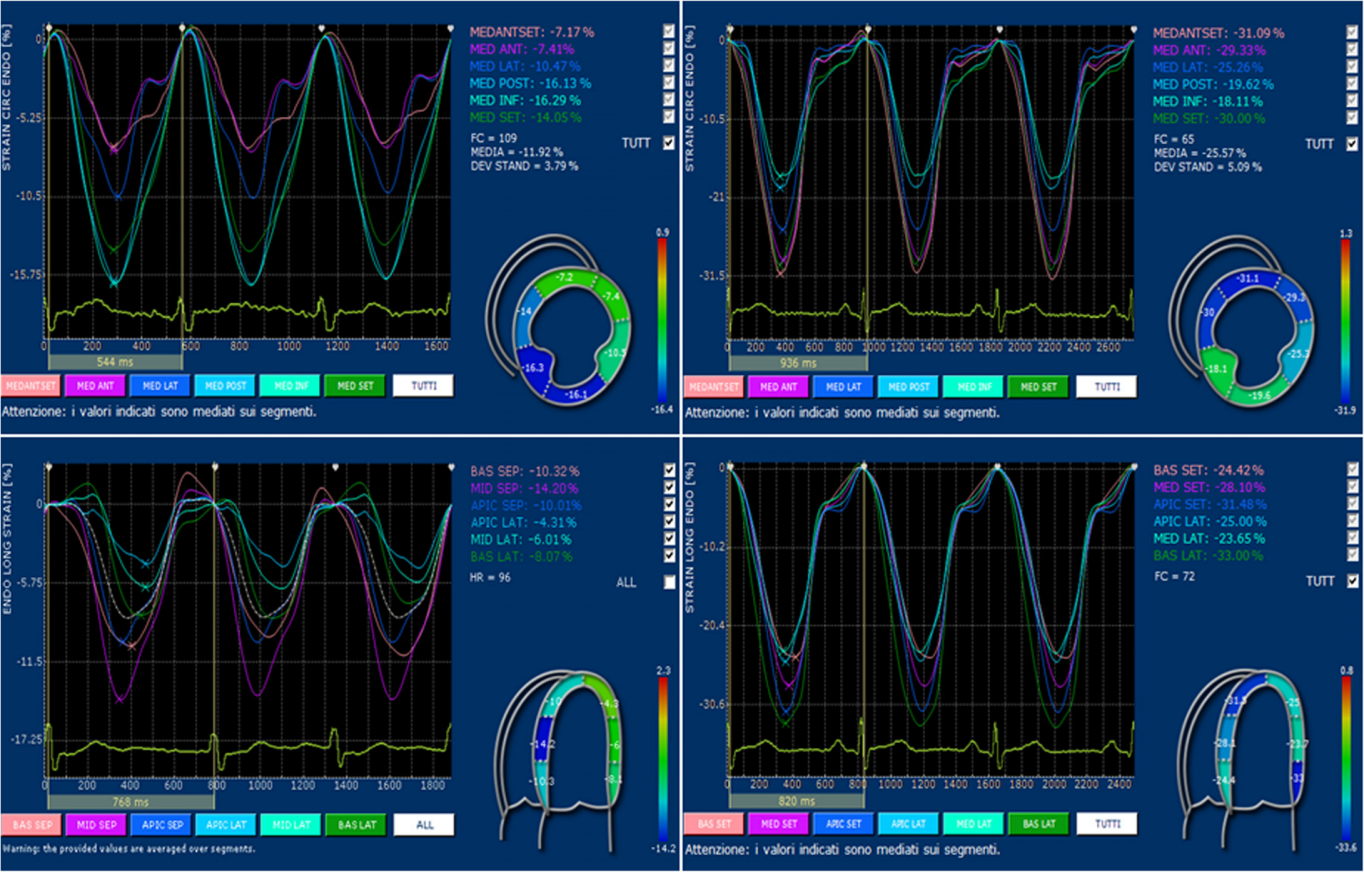
Examples of left ventricular circumferential strain, from mid-ventricular parasternal short axis view [upper panels], and of longitudinal strain, from 4-chamber apical view [lower panels], in a SSc patient [left] and in an healthy subject [right].

**Figure 2:**
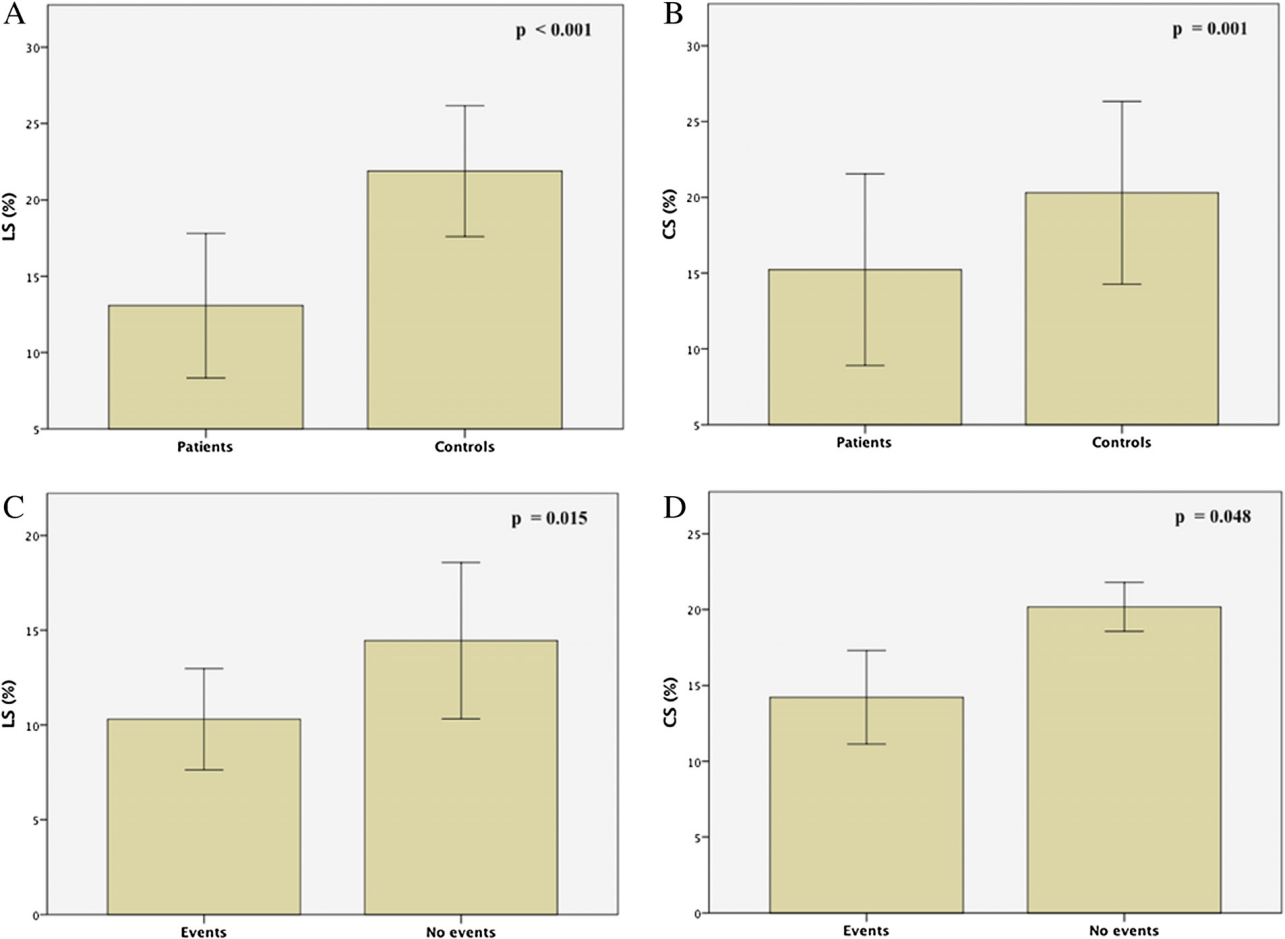
Histograms, with standard deviation bars, of left ventricular longitudinal (LS) and circumferential (CS) strains between patients and controls [upper panels, A and B] and between patients with and without cardiovascular events [lower panels, C and D].

Results of parameters obtained from TDI and 2D strain analysis are listed in Table 3. TDI systolic and diastolic velocities as well as E/e’ ratio were comparable between patients and controls; on the other hand, CS and LS were significantly lower in patients than in healthy subjects (p = 0.001 and p < 0.001 respectively) [Figure 1; Figure 2, upper panels], whereas no significant differences were observed with respects to RS.

**Table 3:** Left ventricular tissue Doppler imaging velocities and global strains in controls and patients

	**Controls (n=30)**	**SSc (n=29)**	**p**
**Septal s’, cm/s**	8.4±0.3	8.0±0.3	ns
**Septal e’, cm/s**	9.5±1.5	8.9±1.7	ns
**Septal a’, cm/s**	9.7±1.2	9.6±1.5	ns
**E/e’ ratio**	6.8±1.3	6.9±1.4	ns
**RS, %**	38.5±9.3	35.5±8.1	ns
**CS, %**	−20.4±5.6	−15.3±6.2	0.001
**LS, %**	−22.6±4.1	−13.1±4.8	< 0.001

*RS, CS* and *LS*: radial, circumferential and longitudinal strain, respectively.

**Figure 3:**
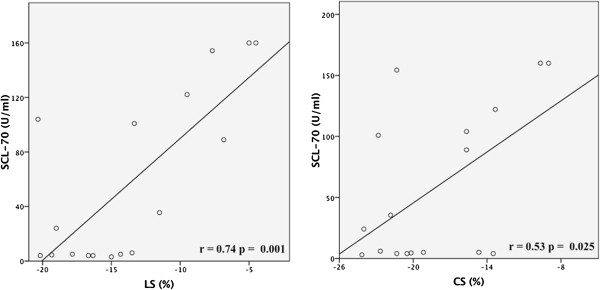
Linear regression lines between longitudinal strain (LS) and Scl-70 antibodies serum levels [left panel] and between circumferential strain (CS) and Scl-70 antibodies serum levels [right panel].

In addition, in the subgroup of patients resulted positive to Scl-70 antibodies (58% of overall group of patients), a significant linear correlation between serum levels of Scl-70 antibodies and both LS and CS was found (r = 0.74 p = 0.001 and r = 0.53 p = 0.025, respectively) [Figure 3].

Follow-up information was available for 26 (89%) of 29 patients and 22 (73%) of 30 healthy subjects. Mean follow-up time was 20±4 months. During follow-up, the predefined cardiovascular events were reached in 12 patients with the occurrence of atrial fibrillation in 6 patients (50%) and symptoms/signs of heart failure (fatigue, dyspnoea, peripheral oedema) in the remaining 6 patients (50%); no cardiovascular events were observed in healthy subjects. As shown in Table 4, no significant differences, with respects to the occurrence of events, were found regarding age, prevalence of main cardiovascular risk factors and standard echocardiographic parameters. On the other hand, as summarized in Table 5, despite a not significant trend for lower TDI velocities and higher E/e’ ratio in patients showing events during follow-up, only LS and CS were significantly reduced in patients with events when compared to the others [Figure 2, lower panels]. Furthermore, in patients with cardiovascular events, a greater prevalence of positivity to Scl-70 antibodies (83%) with, in addition, markedly increased values of serum levels of Scl-70 antibodies were found (p = 0.03 and p = 0.047, respectively).

**Table 4:** General characteristics and standard echocardiographic parameters in patients with and without cardiovascular events during follow-up

	**Events (n=12)**	**No events (n=14)**	**p**
**Age, yrs**	60±2	63±4	ns
**SBP, (mmHg)**	128±9	124±12	ns
**DBP, (mmHg)**	76±10	73±8	ns
**Diabetes, n (%)**	2 (16)	1 (7)	ns
**Dyslipidemia, n (%)**	4 (33)	6 (50)	ns
**Smoking, n (%)**	1 (8)	1 (7)	ns
**Scl-70 antibodies, n (%)**	10 (83)	4 (28)	0.03
**Serum levels of Scl-70 antibodies, U/ml**	83.9±57.6	17.1±41.7	0.047
**LV EDV, ml**	85±7	82±6	ns
**LV ESV, ml**	34±3	30±4	ns
**LV EF, %**	62±3	65±2	ns
**LV Mass, (g/m2)**	72.6±4.3	68.4±3.9	ns
**Mitral E peak velocity, (cm/s)**	63.2±8.5	59.5±9.4	ns
**Mitral A peak velocity, (cm/s)**	62.1±6.7	67.9±9.2	ns
**Mitral E/A ratio**	1.0±0.1	0.8±0.2	ns
**Mitral E deceleration time, (ms)**	176.4±12	182.7±18	ns
**RVOT Prox, mm**	30.9±2.9	30.1±2.8	ns
**RVD1, mm**	33.7±2.6	32.9±2.4	ns
**TAPSE, mm**	20.9±3.1	21.8±2.8	ns
**RV FAC, %**	46.3±5.8	47.1±5.4	ns
**sPAP, (mmHg)**	38.5±3	36.4±7	ns

*SBP and DBP*: systolic and diastolic blood pressure, respectively. *LV*: left ventricular; *EDV and ESV*: end-diastolic and end-systolic volumes, respectively; *EF*: ejection fraction; *RVOT prox*: right ventricular outflow tract proximal diameter; *RVD1*: right ventricular inflow diameter at basal level; *TAPSE*: tricuspid annular plane systolic excursion; *FAC*: fractional area change; *sPAP*: systolic pulmonary arterial pressure.

**Table 5:** Left ventricular tissue Doppler imaging velocities and global strains in patients with and without cardiovascular events during follow-up

	**Events (n=12)**	**No events (n=14)**	**p**
**Septal S’, cm/s**	7.8±0.2	8.1±0.1	ns
**Septal E’, cm/s**	8.6±0.9	9.0±0.6	ns
**Septal A’, cm/s**	9.3±1.0	9.8±1.1	ns
**E/e’ ratio**	7.3±1.2	6.7±1.4	ns
**RS, %**	30.1±5.8	33.2±3.3	ns
**CS, %**	−14.2±3.1	−20.1±1.6	0.048
**LS, %**	−10.3±2.5	−14.4±4.1	0.015

*RS, CS and LS*: radial, circumferential and longitudinal strain, respectively.

### Reproducibility

We obtained good intra-observer and inter-observer reproducibility in the measurement of LS (−1.3±6.2% and −2.4±6.8%, respectively), CS (−1.7±4.6% and −3.1±6.2%, respectively) and RS (4.7±12% and 5.1±17%, respectively).

## Discussion

LV myocardial involvement, even though subclinical and, thus, frequently underestimated, is associated to a worse prognosis of the disease, leading to increased risk of cardiovascular complications in SSc patients [14,15]. Since standard echocardiography, with the assessment of LVEF and PW Doppler mitral inflow parameters, proved to be, in various diseases and SSc as well, often inadequate to identify early myocardial abnormalities, other diagnostic tools are needed in order to detect the onset of cardiac involvement [16-18]. In this respect, TDI and, more recently, 2D strain, have been shown to provide a more accurate evaluation of myocardial function.

The main findings of the present study are: 1) the impairment of both LS and CS, detectable only by means of 2D strain, in asymptomatic SSc patients in the absence of significant differences regarding other echocardiographic parameters, including TDI, in comparison with healthy subjects; 2) a significant correlation of both LS and CS with serum levels of Scl-70 antibodies; 3) a greater impairment of LS and CS in patients showing cardiovascular events during follow-up; 4) higher values of serum levels of Scl-70 antibodies in patients with cardiovascular events.

Along with multiple reports of RV abnormalities in SSc, usually secondary to vascular and interstitial lung disease resulting into pulmonary hypertension [19,20], a primary LV involvement related to organic and functional alterations of microvasculature, including reduced coronary flow reserve, has been observed [21-24]; in support of these data, studies performed using single photon emission computerized tomography (SPECT) have shown multiple ventricular perfusion defects in the absence of coronary artery disease documented by angiography, particularly in patients with pulmonary hypertension [25,26]. Owing to this vasculopathy, a “patchy” myocardial fibrosis develops, with a distribution unrelated to coronary vessels territories [27-30], leading, as consequences, to multiple LV diastolic and systolic abnormalities including lower TDI velocities and impaired myocardial deformation [31,32]. In this respect, a direct relation between myocardial reflectivity, evaluated by integrated cardiac ultrasound backscatter, and LV deformation parameters was found by Mele et al. [33], with a more pronounced impairment of myocardial function in patients with diffuse SSc with respects to the limited form of disease, thus, confirming the pivotal role of myocardial fibrosis in cardiac involvement in SSc. Similarly, previous studies have shown, particularly in diffuse SSc, various myocardial function abnormalities including lower TDI velocities and impaired strain despite normal standard echocardiographic parameters [34,35]. In this respect, TDI, although characterized by excellent temporal resolution, presents some limitations including angle dependency, the impossibility to distinguish passive from active motion and arbitrary choice of regions of interest [36]; on the other hand, 2D strain, even though currently limited by a still lacking standardization of reference values particularly due to inter-vendor variability, proved to be more sensitive and accurate, than TDI, in the assessment of myocardial deformation and, therefore, in the detection of early myocardial abnormalities in various diseases [37,38]. Our findings support these data, as we observed impaired LS and CS in spite of preserved other echocardiographic parameters including TDI velocities. Similarly, Spethmann et al. [39] reported, in SSc patients with normal LVEF, an impairment of LV global longitudinal strain and strain rate, assessed by use of speckle-tracking echocardiography, with, especially, a reduced deformation of basal myocardial segments. Furthermore, an association of impaired LS and CS with lower values of peak VO2 and abnormal Holter electrocardiographic findings has been, previously, observed in SSc patients [40]. In this respect, the impairment of LV longitudinal deformation may reflect, despite preserved LVEF, the early onset of cardiac involvement, since longitudinal subendocardial fibers are known to be, particularly, vulnerable to myocardial damage and ischemia. Furthermore, in patients with more advanced LV myocardial involvement, an impairment of CS, in addition to abnormal longitudinal deformation, may occur, as found in our study.

We observed, moreover, particularly impaired LS and CS in patients with cardiovascular events during follow-up, whereas, interestingly, no significant differences, with respects to the occurrence of cardiovascular events, were found regarding other echocardiographic parameters, such as LVEF, diastolic mitral inflow parameters, RV measurements and TDI velocities. In this respect, so far, to the best of our knowledge, only in our study, cardiovascular events have been found to be associated with impaired LV longitudinal and circumferential myocardial deformations. These data, together with other results, obtained by Hinchcliff et al. [41], concerning the association between LV TDI velocities and increased risk of death in SSc, suggest that new echocardiographic techniques, such as 2D strain, may be useful not only in the detection of early LV myocardial involvement in SSc but also in the identification of patients at greater risk of cardiovascular events. In this regard, the early diagnosis of cardiovascular involvement in these patients may be particularly valuable in order to provide a more adequate therapeutic management, such as the beginning of cardioprotective drugs including inhibitors of renin-angiotensin-aldosterone system and beta-blockers, even though this therapeutic approach has not been widely investigated, so far, in such a clinical setting.

In addition, we found a significant relation between the impairment of LS and CS and higher serum concentrations of Scl-70 antibodies, with particularly lower values of myocardial deformation at very high serum levels of antibodies. Moreover, very high values of Scl-70 antibodies serum levels were observed in patients showing cardiovascular events. These findings support the prognostic relevance of the abnormalities of LV myocardial deformation in these patients, since Scl-70 antibodies, found in about 15–20% of patients, portend a poorer clinical outcome of the disease, as widely reported [42]. Although reports have been, so far, pointed out on the association of these antibodies with restrictive lung disease, pulmonary hypertension and RV functional abnormalities [43], to date a relation between Scl-70 antibodies and LV involvement in SSc, to the best of our knowledge, has not been, previously, described.

## Limitations

As a limitation of our study, we enrolled a relatively small number of patients. This depends on the low prevalence of SSc (estimated between 3 and 24/100.000) [44]; the size of our population, however, doesn’t differ from that reported in previous studies on this topic. In addition, we could not directly evaluate myocardial fibrosis, by use of cardiac MRI, ultrasound calibrated integrated backscatter or myocardial biopsies, and, thus, we can only hypothesize a relation between fibrosis and the cardiac abnormalities observed in our study. Moreover, as a further limitation of our study, we obtained follow-up information of subjects from telephonic interviews and, thus, we could not perform clinical and echocardiographic examination at the time of the occurrence of cardiovascular events.

## Conclusions

Early impairment of LV function, even though associated to a worse clinical course of SSc, is, frequently, subclinical and hardly detectable by means of standard echocardiography. New echocardiographic techniques, such as, particularly, 2D strain, facilitate the detection of early LV function abnormalities occurring in SSc and, moreover, can be helpful in the identification of patients at increased risk of cardiovascular events. Thus, 2D strain may be a useful diagnostic tool, in addition to standard echocardiography, in the routine evaluation of SSc patients in order to provide a more adequate management of these patients with particular regards to the prevention of the occurrence of cardiovascular complications.

## Competing interests

The authors declare that they have no competing interests.

## Authors’ contribution

MCP performed measurements and statistical analysis, and, in addition, played a major part in the writing of the manuscript. GianlB investigated patients. CZ, GO and GDB took part in reviewing the manuscript. SC and GianfB planned the study, reviewed and discussed the manuscript. All authors have read and approved the final manuscript.
